# Evaluation of macular visibility through a high-order aspheric intraocular lens using a simulated model eye

**DOI:** 10.1097/MD.0000000000031018

**Published:** 2022-10-14

**Authors:** Yuji Yoshikawa, Junji Kanno, Kei Shinoda, Jun Makita

**Affiliations:** a Department of Ophthalmology, Saitama Medical University, Iruma, Saitama, Japan.

**Keywords:** extended depth of focus, multifocal intraocular lenses, contrast, second order spherical aberrations, wide-angle system

## Abstract

We evaluated the macular visibility of a newly designed extended depth of focus (EDOF) intraocular lenses (IOL) using a wide viewing system for macular manipulation (Risight;60D, Carl Zeiss Meditec AG) in a model eye and compared it with various other types of IOLs. We used a model eye that was constructed based on the Glustrand model to compare a newly designed EDOF IOL (DIB00V; Johnson & Johnson Surgical Vision), an EDOF IOL with a diffraction grating (ZXR00V; Johnson & Johnson surgical Vision), and a monofocal aspheric (DCB00V; Johnson & Johnson Vision, XY-1; HOYA Surgical Optics, Tokyo, Japan) or spherical IOL (NX70s; Santen Pharmaceutical Co., Ltd). In the model eye, a 1951 United States Air Force (USAF) test was placed at the location of the macula. The contrasts in a range of spatial frequencies were quantified using the images obtained from the 1951 USAF test target. The contrast at each spatial frequency was plotted and integrated to calculate the area under the curve contrast (AUC-contrast). Qualitative evaluations showed that good-quality images were obtained for all IOLs. At a spatial frequency of 16 LP/mm, the average contrast was the highest for the DIB00V and NX70s (0.216 each). The highest average contrast at 32 LP/mm was obtained using the NX70s (0.128), and at 64 LP/mm using the DIB00V (0.123). The horizontal AUC-contrast was the highest for the NX70s (8.754), and the vertical AUC-contrast was the highest for the DIB00V (8.334). On average, the DIB00V had the highest AUC-contrast value (8.227). The high-order aspheric IOL, DIB00V, was found to exhibit good macular visibility despite being an EDOF IOL.

## 1. Introduction

Multifocal intraocular lenses (IOLs) reduce the need for postoperative eyeglasses. However, they have the negative side effect of halo glare vision and can reduce contrast sensitivity.^[1]^ Therefore, implantation of multifocal IOLs may need to be performed with caution.

The extended depth of focus (EDOF) IOL uses a diffractive structure to extend the depth of focus and improve vision at intermediate distances, as compared to conventional monofocal IOLs.^[2]^ A newly designed EDOF IOL, the TECNIS Eyhance (DIB00V, Johnson & Johnson Surgical Vision; Santa Ana, CA), extends the depth of focus by adding a high-order aspheric surface in the center instead of using a diffractive structure.^[3–5]^ The DIB00V can be used as a monofocal IOL in Japan, and it may become popular in the future because of its lower cost. However, patients with newly designed EDOF IOLs who have or later develop retinal disease may require vitrectomy.

Multifocal IOLs can reduce fundus visibility during vitrectomy.^[6]^ For the macular area in particular, a corneal contact lens provides better retinal image contrast and resolution, magnified images, and better stereopsis, which are preferable for macular manipulation. This may not be the case for eyes with multifocal IOLs, because the retinal image contrast decreases at the joining and diffractive sites.^[6]^ For manipulation in the peripheral area, a non-contact wide-angle lens can be used to ensure a certain degree of visibility, but a non-contact lens reduces macular visibility and retinal image contrast.^[7]^

We evaluated the macular visibility of the newly designed EDOF IOL, DIB00V, using a non-contact wide-angle lens (60D) and a simulated eye and compared it with another aspheric IOL, a spherical IOL, and an EDOF IOL with a diffraction grating.

## 2. Method

**Figure 1.**
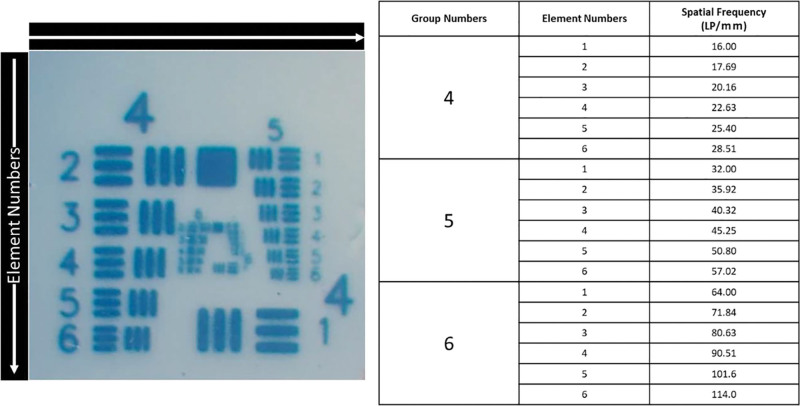
We showed the image of 1951 United States Air Force test target and the corresponding spatial frequency for each stripe are shown.

This study was waived from ethical review because it is not a study involving patients or animals. We used the model eye described in previous reports by Inoue et al^[8,9]^ The simulated eye was constructed based on Glustrand’s model of the human eye, with an axial length of 24 mm, pupil diameter of 7.0 mm, and corneal spherical aberration of + 0.22 µm. In the model eye, a stripe index from the 1951 United States Air Force (USAF) test (Edmund Optics, Barrington, NJ) was placed at the location of the macula. The stripe indices of different groups and elements were printed on the USAF chart, and the spatial frequency corresponding to each index was determined (Fig. 1). The experiment was performed at room temperature, and the model eye was filled with distilled water. All IOLs had the same spherical power (+21.0 diopter) and were centered on the optical axis of the simulated eye.

The IOLs used were as follows: the aspheric IOLs, TECNIS Optiblue (DCB00V; Johnson & Johnson Surgical Vision, Santa Ana, CA), and Vivinex (XY-1; HOYA Surgical Optics, Tokyo, Japan); newly designed EDOF IOL (the higher-order aspheric lens), TECNIS Eyhance (DIB00V; Johnson & Johnson Surgical Vision, Santa Ana, CA); EDOF lens with a diffraction grating, TECNIS Symfony (ZXR00V; Johnson & Johnson Surgical Vision, Santa Ana, CA); and spherical lens, Eternity Fine Natural (NX70s, Santen Pharmaceutical Co., Ltd., Osaka, Japan).

A Lumera 700 surgical microscope (Carl Zeiss Meditec, Jena, Germany) with a True Vision 3D Visualization System for ophthalmology (Leica Microsystems, Wetzlar, Germany) was used to perform the experiments. The illumination was delivered using a 25G Chandelier Constellation lighting system (Alcon Laboratories, Geneva, Switzerland) at a light intensity of 25%. The True Vision 3D Visualization System was set to 46.8 brightness, 53.9 contrast, 1.1 gamma, 2 gain, 90 saturation, 0 hue, and 70% aperture. A wide viewing system for macular manipulation (Risight; 60D, Carl Zeiss Meditec AG) was used for macular observation. The images recorded by the True vison 3D Visualization System are stored in a side-by-side format. Thus, images viewed by the right eye were used for analysis.

The obtained images of the 1951 USAF test target were imported into Image J software (National Institutes of Health, Bethesda, MD), and the contrast at each spatial frequency was quantified. The contrast was calculated as (I_max_-I_min_)/(I_max_ + I_min_) using the highest (I_max_) and lowest (I_min_) signal intensity values for each stripe index.^[8,9]^ The contrast at each spatial frequency was plotted and integrated to calculate the area under the curve contrast (AUC-contrast).

## 3. Results

**Figure 2.**
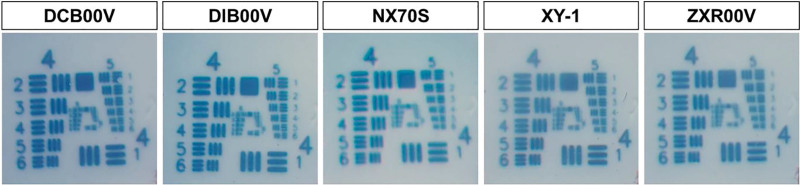
Images of 1951 U.S. Air Force test targets through each IOL are shown. IOL = intraocular lens.

Figure 2 shows the results of the images for each IOL. Qualitative evaluation showed that good-quality images were obtained with all IOLs, regardless of whether they were spherical, aspheric, monofocal, or EDOF IOLs.

**Figure 3.**
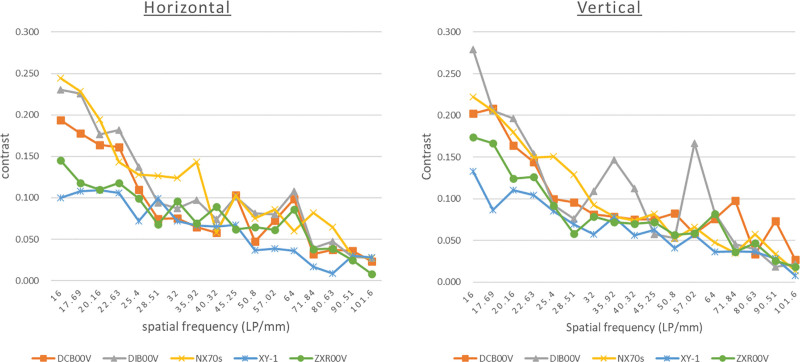
We show a line graph plotting the contrast at each spatial frequency. Both vertically and horizontally, the contrasts of the DIB00V and NX70s were high, while the contrasts of the XY-1 and ZXR00V tended to be low.

As shown in Table 1, at a spatial frequency of 16 LP/mm, the average contrast was the highest for the DIB00V and NX70s at 0.216 each. The highest average contrast at 32 LP/mm was obtained using the NX70s (0.128) and at 64 LP/mm using the DIB00V (0.123). Figure 3 shows a plot of the contrast at each spatial frequency for the vertical and horizontal stripes.

**Table 1:** Contrast values at typical spatial frequencies and area under the curve-contrast values.

		DCB00V	DIB00V	NX70S	XY-1	ZXR00V
Spatial frequency (LP/mm)						
Horizontal	16	0.207	0.216	0.200	0.194	0.171
	32	0.075	0.094	0.127	0.099	0.068
	64	0.073	0.080	0.086	0.039	0.062
Vertical	16	0.169	0.215	0.233	0.155	0.170
	32	0.095	0.076	0.129	0.070	0.058
	64	0.058	0.167	0.066	0.057	0.058
Average	16	0.188	0.216	0.216	0.175	0.170
	32	0.085	0.085	0.128	0.084	0.063
	64	0.066	0.123	0.076	0.048	0.060
AUC						
Horizontal		7.019	8.219	8.754	4.793	6.212
Vertical		8.067	8.334	7.293	5.104	6.306
Average		7.543	8.277	8.024	4.949	6.259

AUC = area under the curve.

Both vertically and horizontally, the contrasts of the DIB00V and NX70s were high, while the contrasts of the XY-1 and ZXR00V tended to be low. The AUC-contrast was the highest in the horizontal for the NX70s (8.754) and the highest in the vertical for the DIB00V (8.334). On average, the DIB00V had the highest AUC-contrast value (8.227).

## 4. Discussion

The newly designed EDOF IOL, a high-order aspherical lens, showed good visibility of the macula when observing the fundus during surgery using a non-contact lens. In addition, the image contrast value of this lens was equal to or higher than that of other spherical and aspheric IOLs. Compared to EDOF lenses with diffraction structures, the good contrast images could be obtained.

Conventional multifocal IOLs, including EDOF lenses, have problems with fundus visibility during vitrectomy. In our study, the image contrast of the ZXR00V, which is a conventional EDOF lens with a diffractive structure, was lower than that of the DCB00V, which is a conventional aspherical IOL and has a similar platform as the ZXR00V (Table 1). However, when comparing the DCB00V with the DIB00V, which is a newly designed EDOF IOL that has a similar platform as the DCB00V, the image contrast value was higher in the DIB00V. Compared to the ZXR00V, the DIB00V has no diffractive structure. So, the fundus visibility might be well maintained. Moreover, second order spherical aberrations are added at the center of the lens.^[3–5]^ So, depth of field was improved and as a result, image contrast may be improved.

The DCB00V and DIB00V aspherical IOLs produced better contrast images than the XY-1. TECNIS IOLs, such as the DCB00V, DIB00V, and ZXR00V, use acrylic material with a high Abbe number.^[10]^ Lenses with a high Abbe number correct chromatic aberrations and provide good contrast; therefore, the material of the IOL might affect the image quality.

Although the multifocal IOLs including EDOF IOLs are thought to be superior than the monofocal IOLs in improving postoperative near-visual acuity, they have a disadvantage of fundus visibility during vitrectomy. However, the newly designed EDOF lens, the DIB00V, was rather surprising in that the EDOF effect favored macular visibility.

In the future, it will be necessary to confirm the reproducibility of image quality and to conduct similar studies with other types of multifocal and EDOF lenses.

We report that a new design of EDOF lens, the DIB00V high-order aspheric lens, with non-contact wide-angle lens shows good macular visibility.

## Author contributions

**Conceptualization:** Yuji Yoshikawa.

**Data curation:** Yuji Yoshikawa, Junji Kannoa.

**Investigation:** Yuji Yoshikawa.

**Supervision:** Kei Shinoda, Jun Makita.

**Writing – original draft:** Yuji Yoshikawa.

**Writing – review & editing:** Kei Shinoda, Jun Makita.
